# 3-Hydroxykynurenine in Regulation of *Drosophila* Behavior: The Novel Mechanisms for *Cardinal* Phenotype Manifestations

**DOI:** 10.3389/fphys.2020.00971

**Published:** 2020-08-07

**Authors:** Aleksandr V. Zhuravlev, Oleg V. Vetrovoy, Polina N. Ivanova, Elena V. Savvateeva-Popova

**Affiliations:** ^1^Laboratory of Neurogenetics, Pavlov Institute of Physiology Russian Academy of Sciences, Saint Petersburg, Russia; ^2^Laboratory of Regulation of Brain Neuron Functions, Pavlov Institute of Physiology Russian Academy of Sciences, Saint Petersburg, Russia; ^3^Department of Biochemistry, Faculty of Biology, Saint Petersburg State University, Saint Petersburg, Russia; ^4^Department of Anatomy and Physiology of Humans and Animals, Faculty of Biology, Herzen State Pedagogical University of Russia, Saint Petersburg, Russia

**Keywords:** *Drosophila*, kynurenine pathway, oxidative stress, spontaneous locomotor activity, 3-hydroxykynurenine, *cardinal*, protein modification by 3-hydroxykynurenine

## Abstract

Dysfunctions of kynurenine pathway of tryptophan metabolism (KPTM) are associated with multiple neuropathologies in vertebrates and invertebrates. *Drosophila* mutants with altered content of kynurenines are model objects for studying the molecular processes of neurodegeneration and senile dementia. The mutant *cardinal* (*cd^1^*) with accumulation of the redox stress inductor 3-hydroxykynurenine (3-HOK) shows age-dependent impairments of the courtship song and middle-term memory. The molecular mechanisms for 3-HOK accumulation in *cd^1^* are still unknown. Here, we have studied age-dependent differences in spontaneous locomotor activity (SLA) for the wild type strain *Canton-S* (*CS*), *cd^1^*, and *cinnabar* (*cn^1^*) with an excess of neuroprotective kynurenic acid (KYNA). We have also estimated the level and distribution of protein-bound 3-HOK (PB-3-HOK) in *Drosophila* brains (Br) and head tissues. The middle-age *cd^1^* show the higher running speed and lower run frequency compared to *CS*, for *cn^1^* the situation is the opposite. There is a decrease in the index of activity for 40-day-old *cd^1^* that seems to be an effect of the oxidative stress development. Surprisingly, PB-3-HOK level in *Drosophila* heads, brains, and head capsules (HC) is several times lower for *cd^1^* compared to *CS*. This complements the traditional hypothesis that *cd^1^* phenotype results from a mutation in phenoxazinone synthase (PHS) gene governing the brown eye pigment xanthommatin synthesis. In addition to 3-HOK dimerization, *cd^1^* mutation affects protein modification by 3-HOK. The accumulation of free 3-HOK in *cd^1^* may result from the impairment of 3-HOK conjugation with some proteins of the brain and head tissues.

## Introduction

Kynurenine pathway of tryptophan metabolism (КPTM) is the major path of tryptophan catabolism in mammals (Badawy, 2017). Leading to nicotinamide adenine nucleotide (NAD^+^) production in cell, KPTM also plays an important role as the source of neuroactive compounds, collectively called kynurenines. The imbalance of kynurenines in nervous system has been observed during neurodegenerative disorders, such as Alzheimer’s, Parkinson’s, and Huntington’s diseases (Schwarcz et al., 2012). The multiple neurotropic effects of kynurenines were shown for both vertebrates and invertebrates (Lapin, 2004). A KPTM metabolite quinolinic acid (QUIN) is an agonist of ionotropic glutamate receptors (iGluR; El-Defrawy et al., 1986). Kynurenic acid (KYNA) is a non-specific iGluR (Kessler et al., 1989) and α7 nicotinic acetylcholine receptor (Hilmas et al., 2001) antagonist. It can interact with both mammalian and *Drosophila* iGluR receptors (Zhuravlev et al., 2012), ameliorating excitotoxicity. 3-hydroxykynurenine (3-НОК) and 3-hydroxyanthranilic acid (3-HAA) inhibit lipid peroxidation (Christen et al., 1990; Zhuravlev et al., 2016). At the same time, 3-HOK oxidative dimerization may cause overproduction of reactive oxygen species (ROS) and cell death (Okuda et al., 1996, 1998).

**Figure 1:**
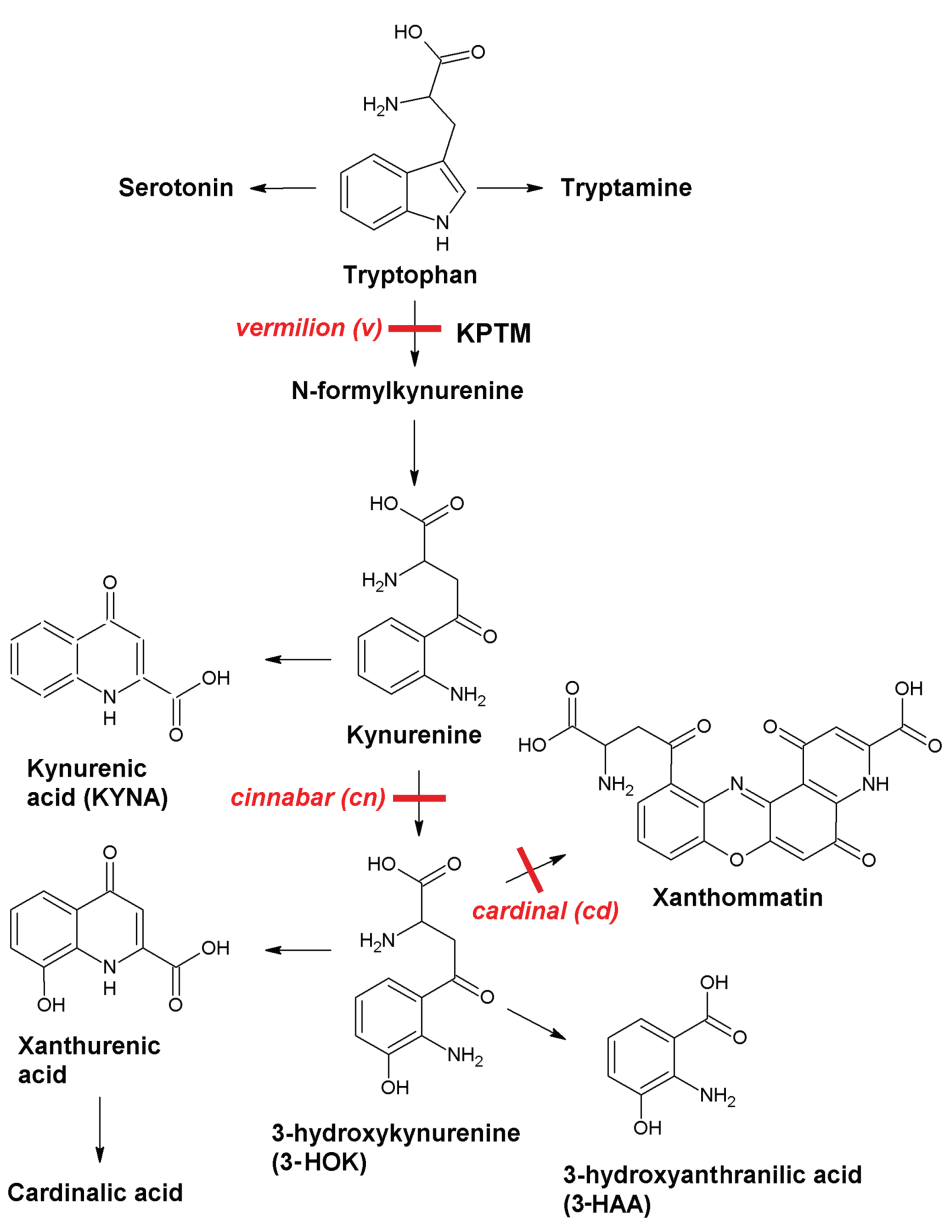
Kynurenine pathway of tryptophan metabolism (KPTM) in *Drosophila*.

Neurotropic effects of kynurenines can be studied using simple model objects, such as *Drosophila melanogaster* mutants (Figure 1). In insects, KPTM is the source of the brown eye pigments ommochromes, including xanthommatin that is synthesized upon 3-HOK dimerization. 3-HOK also produces xanthurenic and cardinalic acids. In *vermilion* (*v*), KPTM is blocked with six-fold rise of tryptophan level. In *cinnabar* (*cn*), the inactivation of kynurenine monooxygenase (KMO) gene leads to two-fold increase in KYNA level. In *cardinal* (*cd*), there is a 2.9-fold rise of 3-HOK level, due to some defects in phenoxazinone synthase (PHS) gene. Xanthommatin can also be produced non-enzymatically due to the oxidative 3-HOK autodimerization. QUIN and nicotinic acid are not synthesized from 3-HAA in *Drosophila* and 3-HAA level is low (Linzen, 1974; Savvateeva et al., 2000; and references therein). Hence, *cd* can be used to study the specific neurotoxic effects of 3-HOK. Studying the behavioral characteristics of *Drosophila* KPTM mutants is of particular interest, as *cn* and *cd* alleles are marker genes in many transgenic fly strains, including those used in UAS-GAL4 system.

In many cases, 3-HOK and KYNA manifest themselves as antagonists. The increase in 3-HOK/KYNA level for *Drosophila* mutant *htt*, a model for Huntington’s disease, leads to neurodegeneration (Green et al., 2012). Inhibition of KMO increases 3-HOK/KYNA level, showing an advancement in neurodegeneration (Campesan et al., 2011). In aged *cd*, the middle-term memory is impaired and the calyx neuropathology is observed, while in aged *cn* the synaptic pathology is less (Savvateeva et al., 2000). On day 29, *cd* males demonstrate courtship song defects, such as high variance of interpulse interval (VIPI), while KYNA accumulation in *cn* seems to have neuroprotective effects, as their VIPI is lower compared to *cd* and the wild type *Canton-S* (*CS*; Savvateeva-Popova et al., 2003). For 5-day-old *cd^1^*, memory is impaired by heat shock and is restored by the application of a phenolic antioxidant (Nikitina et al., 2018). Heat shock induces apoptosis in *cd^1^* brain (Br) on day 5 (Savvateeva-Popova et al., 2003), which may be due to acceleration of 3-HOK autoxidation leading to hydrogen peroxide production. The dual role of 3-HOK in oxidative stress development is manifested as total antioxidant capacity decrease in head tissues of 5–29-day-old *cd^1^* compared to *CS* along with the tendency of lipid peroxidation level decrease in *cd^1^* (Zhuravlev et al., 2018).

An important characteristic of *Drosophila* is spontaneous locomotor activity (SLA). Spontaneous activity can be defined as “the observable activity exhibited by an animal when not specifically activated by external stimuli” (Ewing, 1963). Previously, age-dependent SLA for *Drosophila* KPTM mutants was estimated as the percentage of moving females in a group recorded at time intervals (Kamyshev, 1980). However, this method did not consider specific SLA parameters, such as percentage of movement time (index of activity), running speed, total speed, etc. The evaluation of SLA parameters for *Drosophila* KPTM mutants was performed for the third instar larvae (Zakharov et al., 2012). Most of them decrease in *cn^1^* with KYNA excess, while *v^1^* and *cd^1^* have slightly higher running speed compared to *CS*. In this study, we have evaluated the age-dependent changes in SLA parameters for the adult male *CS*, *cd^1^*, and *cn^1^*. We have also performed the measurement of 3-HOK protein-bound form (PB-3-HOK) in flies’ heads, as well as its distribution within *CS* and *cd^1^* brains.

## Materials and Methods

### Materials

#### *Drosophila* Strains

The research was performed on *D. melanogaster* strains from Biocollection of Pavlov Institute of Physiology Russian Academy of Sciences, Saint Petersburg, Russia. The following strains were used:

1. *Canton-S* (*CS*), the wild type strain.
2. *vermilion* (*v^1^*) – a mutation in tryptophan 2,3-dioxygenase (TDO) gene; no kynurenines.
3. *cinnabar* (*cn^1^*) – a mutation in KMO gene; 3-HOK lack, KYNA excess.
4. *cardinal* (*сd^1^*; Bloomington Drosophila Stock Center #3052) – a mutation in PHS gene; 3-HOK excess.

All mutant strains were out-crossed to *CS* for nine generations. The strains were raised on standard yeast–raisin medium at 12:12 (8 a.m.–8 p.m.) daily illumination cycle at 25 ± 0.5°C; humidity was not controlled. Males of the studied strains were collected without narcotization within 1 day after eclosion and kept on the standard medium in small fruit fly vials at 22 ± 0.5°C until the experiment.

### Methods

#### Spontaneous Locomotor Activity

Males were kept in small groups (4–5 males in a vial) before experiment. The same flies were tested at the different ages, with the exception of some dead or flying away. For each KPTM strain and the fly age, the experiment was carried out within a single day (mostly within 11 a.m.–3 p.m.; 18–24 flies per day) at 22 ± 1°C. SLA significantly varied from day to day, so *CS* flies of the same age served a control in each experiment. The procedure of analysis is described in (Zakharov et al., 2012), being adapted for imagoes. Flies were placed separately in flat round neon-lit chambers (diameter 20 mm, height 3 mm). Fly position on the chamber plane was represented by the weighted *X* and *Y* coordinates of all pixels brighter than the threshold level 60. Fly movement was then recorded for 1 h through the transparent сover using Logitech QuickCam web camera. Tracks were analyzed using Locotrack software (Zakharov, 2017). The full record was divided into 1 s quanta, and the mean speed of fly movement in each quantum was calculated. If the result was lower than 1 mm/s, the fly was considered to be in the rest (Colomb et al., 2012), otherwise it was considered to run. All the other settings were default for imagoes. The following SLA parameters were estimated: (1) index of activity (C.U.), (2) run frequency (N/100 s), (3) running speed (mm/s), (4) total speed (mm/s), and (5) run bout time (s). To reveal the differences between *CS* and *cd^1^*, large samplings were tested. For some tracks, the total fly trajectories were graphically visualized.

#### PB-3-НОК Level in *Drosophila* Homogenates

Flies heads or head parts were homogenized on ice in TE buffer (0.2 M tris-HCl, 1 mM EDTA, pH 7.4: 40–50 heads in 600 μl; six brains or head capsules (HC) in 30 μl). The homogenates were centrifuged at 4°C at 1,000 *g* for 10 min. Two microliters of supernatant was immobilized on a nitrocellulose membrane (Protran; Whatman). 3-HOK level was measured using dot blot technique according to the Abcam protocol (Abcam, Cambridge, UK). The membrane was incubated with anti-3-HOK monoclonal antibody (1:2000; Thermo Scientific, #MA1-16616), then with secondary HRP-conjugated antibody (1:1000, Thermo Scientific, #31431), and stained using Vector NovaRed Peroxidase Substrate Kit (Vector Laboratories). The signal level was estimated using Fiji software (Schindelin et al., 2012). Signal was normalized to β-actin (primary antibody: 1:2000; Sigma-Aldrich, A2066; secondary antibody: 1:1000; Abcam, ab97064) or to the total protein level measured by amido black staining (Sigma-Aldrich, 1.01167). In a control experiment, the homogenates were purified from proteins using Microcon Ultracel YM-3 columns (Millipore), and no signal was observed. Thus, the detected 3-HOK form is a protein-bound form (PB-3-HOK).

According to antibody manufacturer’s, BSA-3-HOK conjugates were used as standards. To perform conjugation, LC-SPDP crosslinker (Thermo Scientific, #21651) and L,D-3-HOK (Sigma-Aldrich, H1771) were mixed in PBS-EDTA buffer (pH 7.5), incubated for 60 min at room temperature and mixed with BSA (final concentrations: in 1 ml: 0.5 mM LC-SPDP, 0.95 mM (0.2 μg/μl) 3-HOK, 0.2% BSA). The mixture was incubated overnight at 4°C and centrifuged in spin columns to remove free 3-HOK. Conjugates were diluted in 100 μl PBS-EDTA (~2 μg/μl 3-HOK in conjugate). To make standard curve, serial dilution of standard was prepared, along with dilution of *CS* head capsule homogenate. To test antibody specificity, PB-3-HOK antibody was preincubated overnight with BSA-3-HOK (1:3 v/v ratio), and optical density (OD) difference with and without preincubation was measured.

To check the PB-3-HOK mass range, homogenates were mixed with Laemmly buffer (without 2-mercaptoethanol), separated by sodium dodecyl sulfate-polyacrylamide gel electrophoresis (SDS-PAGE), transferred to the PVDF membrane (Thermo Fisher Scientific, USA), and stained as it mentioned above.

#### PB-3-НОК Distribution in *Drosophila* Brains

The experiments were performed according to (Wu and Luo, 2006) with some modifications. After fixation in 4% paraformaldehyde solution in PBS buffer for 30 min, brains were incubated with primary antibody to 3-HOK (1:100 in PBT buffer) for 5 days and then with AlexaFluor 568 secondary antibody (Invitrogen, #11037; 1:100) for 1 day with shaking at 4°C. FITC-conjugated anti-horseradish peroxidase antibody (1:100; Jackson ImmunoResearch, 123-095-021) was used as a neuronal marker. Nuclei were stained with DAPI (1.2 μg/ml in PBS). The brains were scanned frontally by the confocal laser scanning microscope (LSM 710 Carl Zeiss; Confocal microscopy Resource Center; Pavlov Institute of Physiology Russian Academy of Sciences, Saint Petersburg, Russia) using X20 objective and analyzed with Fiji. For each brain, the average intensity of PB-3-HOK staining was measured in one hemisphere within the darkened area of pedunculus (Ped; *x*) and the area of the same size above it (*y*). *x* and *y* values were averaged for three optical slices with maximum Ped dimming. *R* = *x*/*y* – the relative decrease in PB-3-HOK for Ped area.

#### Statistical Analysis

Statistical analysis was performed with the help of Social Science Statistics resource^1^ using two-tailed Mann-Whitney U test (for SLA parameters) or two-tailed *t*-test (for PB-3-HOK level estimation).

## Results

### Spontaneous Locomotor Activity

**Figure 2:**
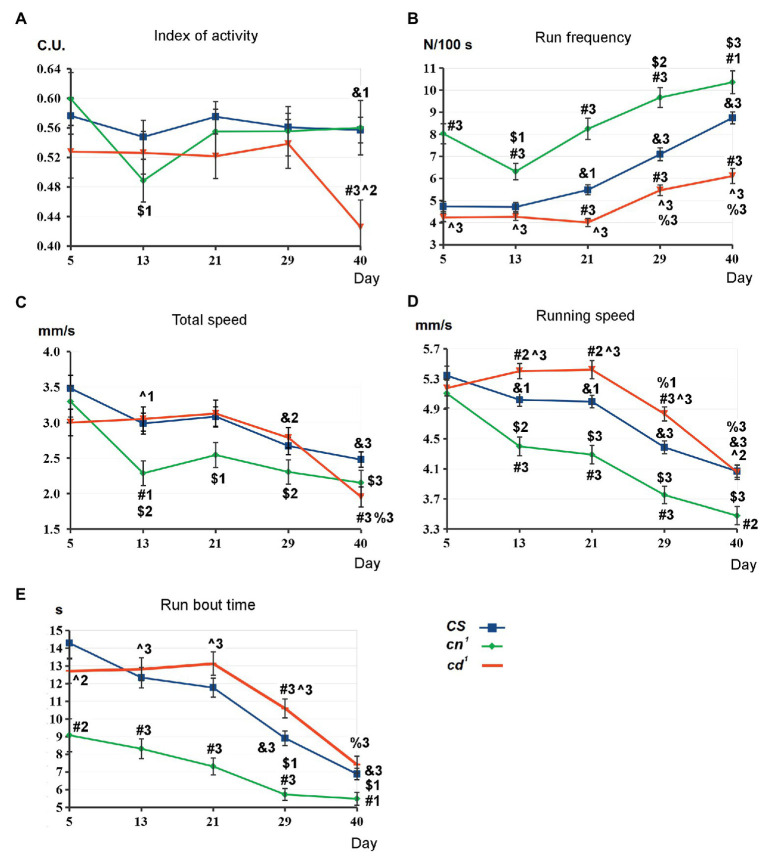
Spontaneous locomotor activity (SLA) parameters for kynurenine mutants. Statistical differences: # from *Canton-S* (*CS*), ^ from *cinnabar* (*cn^1^*), & from 5-day-old *CS*, $ from 5 day-old *cn^1^*, and % from 5 day-old *cardinal* (*cd^1^*). Method of analysis: two-tailed Mann-Whitney U-test, *p* < 0.05 (1), *p* < 0.01 (2), *p* < 0.001 (3); for *CS*: *n* = 163 (day 5)–143 (day 40), the average data of seven independent experiments; for *cn^1^*: *n* = 47 (day 5)–32 (day 40), the average data of two independent experiments; for *cd^1^*: *n* = 118 (day 5)–108 (day 40), the average data of five independent experiments. Here and below: mean values with standard error are shown **(A-E):** see in the text.

The comparative analysis of SLA parameters has been performed for *CS*, *cd^1^*, and *cn^1^* (Figure 2; Supplementary Figure S1). There are no interstrain differences on day 5. Index of activity is similar for all strains, except its decrease in *cd^1^* compared to *CS* on day 40 (A). For *cd^1^*, run frequency is higher than for *CS* on days 21–40 (B). Total speed is the same for the both strains except its decrease in *cd^1^* on day 40 (C), while running speed is higher in *cd^1^* on days 13–29 (D) and run bout time is higher on day 29 (E). For *cn^1^*, the picture is actually the opposite: There is a pronounced increase in run frequency compared to *CS* during the studied period, along with decrease in total speed, running speed, and run bout time after day 5.

*CS* does not show the age-dependent changes in index of activity up to day 40. For all strains, run frequency rises with aging after day 13 that means the increase in the number of stops. Running speed and run bout time decrease for all strains on days 29–40 compared to day 5. For *cn^1^*, there is a slight decrease in several SLA parameters on day 13. However, we also observed a similar decrease in the control *CS* group, so it is possibly explained by the local change of flies’ activity due to some external factors. The pronounced decrease in index of activity for 40-day-old *cd^1^* compared to *CS* and 5-day-old *cd^1^* seems to be the effect of the neurotoxic processes.

*cn^1^* index of activity does not differ from *CS* on day 40. Hence, it is not affected by the lack of the eye pigments ommochromes in *cn^1^*. The form of tracks shows centrophobia for all strains (Supplementary Figure S2A), as flies prefer to move along the walls of the chamber and avoid the central space, in agreement with previous data (Colomb et al., 2012). Most of the tracks form a complete circle demarcating the chamber edge, as flies mostly move along the side wall of the chamber. To test the influence of the pigment synthesis defect on flies’ locomotion, we analyzed their SLA under the red light on day 1 (Supplementary Figure S2B). Centrophobia is more expressed here, and some tracks are incomplete. Though both 1-day-old *cn^1^* and *cd^1^* lack xanthommatin (bright red eyes), a significant difference in their running speed was observed, indicating it to be the most specific parameter that depends solely on 3-HOK/KYNA level for each strain but not on their visual abilities.

### Protein-Bound 3-HOK Level and Distribution

**Figure 3:**
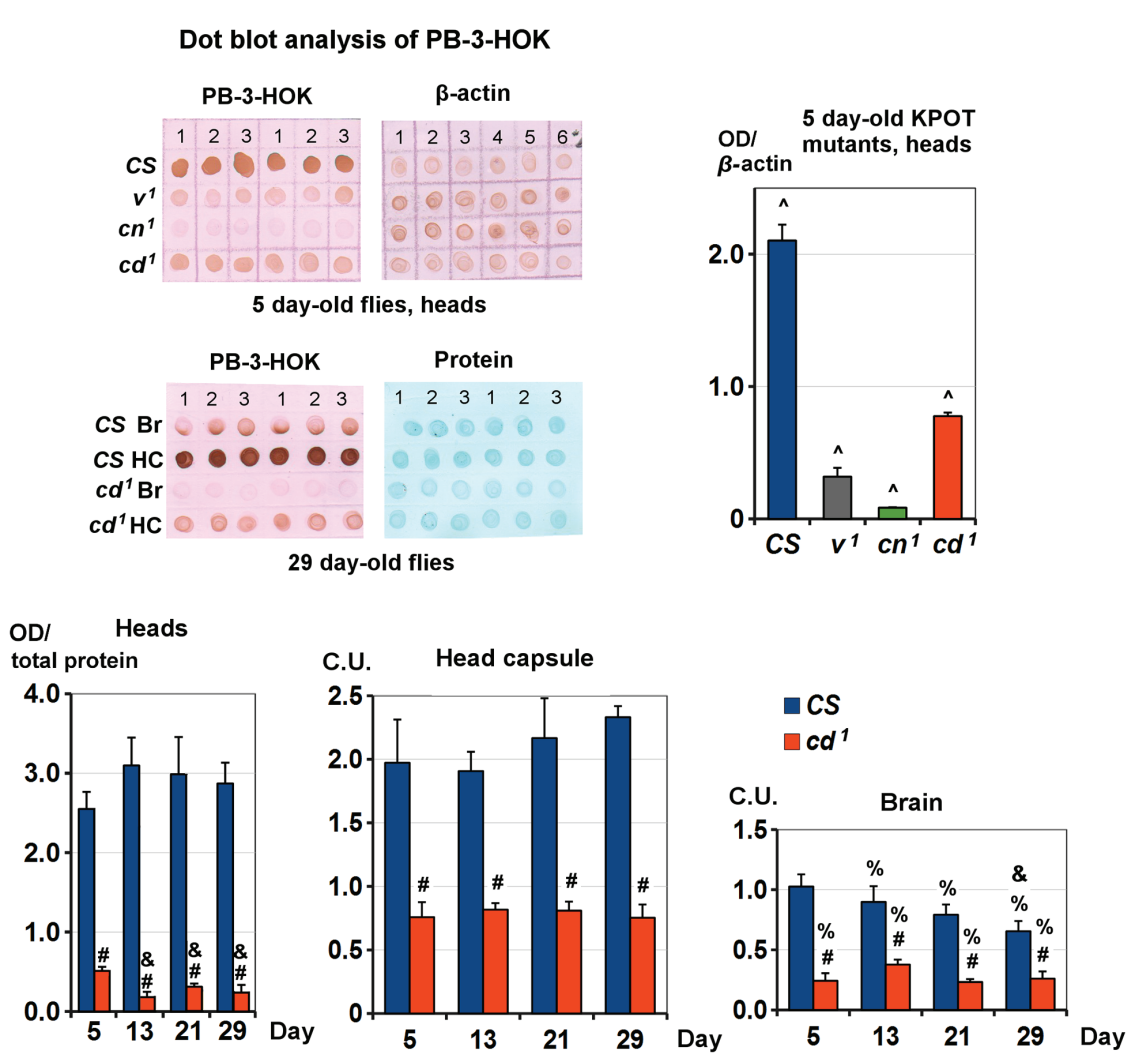
Protein-bound 3-HOK (PB-3-HOK) level in kynurenine mutants heads. Five-day-old *CS* and KPOT mutants: expressed as PB-3-HOK optical density (OD) normalized to beta-actin OD. All interstrain differences (^) are statistically significant (two-tailed *t*-test; *p* < 0.05, *n* = 3). *CS* and *cd^1^* heads: expressed as OD normalized to total protein OD. Statistical differences: # from *CS* (*p* < 0.01), & from 5-day-old flies of the same strain (*p* < 0.05; two-tailed *t*-test; *n* = 5). *CS* and *cd^1^* brains (Br) and head capsules (HC): expressed as OD normalized to total protein OD and to the mean of the all values for a given age (C.U.). Statistical differences: # from *CS*, & from 5-day-old flies of the same strain, and % from head capsules (two-tailed *t*-test, *p* < 0.05; *n* = 3).

Non-specific lens proteins modification by 3-HOK seems to be involved in cataract formation (Korlimbinis and Truscott, 2006). To check possible changes of 3-HOK-protein conjugation in *Drosophila*, PB-3-HOK levels were measured in fly heads, as well as in brains and the head capsules separately. For heads of 5-day-old *Drosophila* KPTM mutants, PB-3-HOK decreases in the following order: *CS* > *cd^1^* > *v^1^* > *cn^1^* (Figure 3). The decrease in PB-3HOK for *cd^1^* compared to *CS* throughout the studied period of life is the most unexpected fact, as the level of free 3-HOK is known to be higher in *cd^1^*. There are no significant age-dependent changes in *CS* PB-3-HOK level; for *cd^1^*, it slightly decreases on days 13–21. This effect occurs simultaneously with the increase of *cd^1^* running speed (see Figure 2). PB-3-HOK level decreases both in brains and in the head capsules of *cd^1^*. Its level in the head capsules is higher compared to brains, except for 5-day-old *CS*. For the studied range of concentrations, there is a linear dependence of PB-3HOK level in homogenate and OD of immunohistochemical staining (Supplementary Figure S3). In the head capsules, 3-HOK is mainly associated with 40–55 kDa proteins, for which nature and biological functions remain unknown. In brains, PB-3-HOK level is too low to be able to detect clear bands.

**Figure 4:**
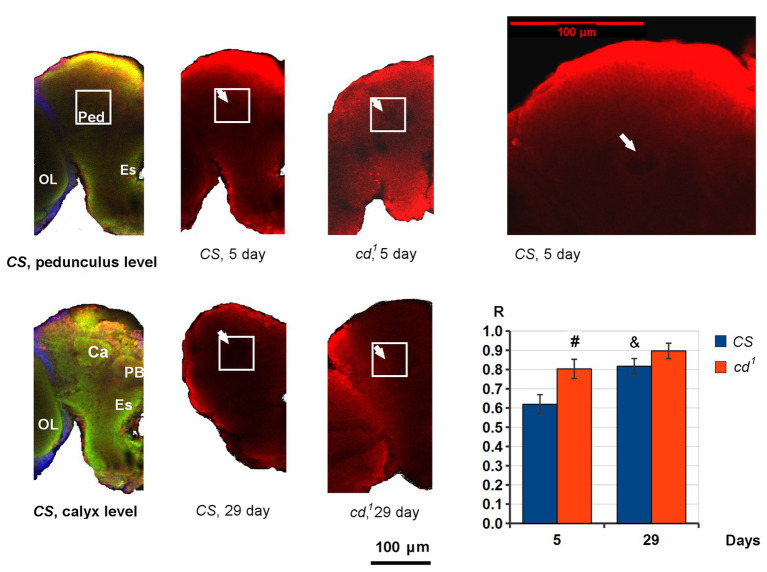
PB-3-HOK distribution in *CS* and *cd^1^* brain. The part of the left hemisphere is shown frontally. The area of interest (white square) is shown on pedunculus (Ped) level. The area of PB-3-HOK decrease in Ped is indicated with a white arrow. Statistical differences: # *cd^1^* from *CS* (*p* < 0.05), and & *CS* day 29 from *CS* day 5 (*p* < 0.01); two-sided *t*-test; *n* = 8 (*CS* day 5), 9 (*CS* day 29), 11 (*cd^1^* day 5), 5 (*cd^1^* day 29). Color scheme: green – neuropile, red – PB-3-HOK, blue – nuclei. Ca, calyx; Es, esophagus; OL, optic lobe; PB, protocerebral bridge; Ped, pedunculus.

PB-3-HOK distribution in *Drosophila* brains is nearly uniform. However, in 5-day-old *CS*, we can see the decline in PB-3-HOK level within a small round area just below the calyx of the mushroom body, where Ped begins (Figure 4). *R* value shows the normalized PB-3-HOK level within this area. On day 5, *R* is higher for *cd^1^* and the area of decline is not observed so clearly as in *CS*. On day 29, *R* significantly increases for *CS* compared to day 5, and for *cd^1^* the darkened area is virtually absent (R → 1). In some brains, the darkened area is closer to the antennal lobe; it also can vary in size and shape. Possibly, its lower contrast for *cd^1^* is due to the lower total brain PB-3-HOK level for this strain.

## Discussion

Assessment of specific SLA parameters for *Drosophila* mutants unravels genes controlling the motor functions (Fedotov et al., 2014) and helps to understand the molecular mechanisms of kynurenines’ neurotropic activity (Zakharov et al., 2012). As we have shown here for 13–29-day-old *cd^1^*, running speed is higher and run frequency is lower compared to *CS*. Stimulating effect of 3-HOK excess on the running speed may be caused by the rise in ROS level, as total antioxidant capacity decreases in *cd^1^* heads (Zhuravlev et al., 2018). Decreased level of mitochondrial hydrogen peroxide in transgenic *Drosophila* leads to decrease in walking speed, as well as lifespan, indicating the importance of free radicals production at moderate level for the life processes (Bayne et al., 2005). Total SLA decrease in 40-day-old *cd^1^* may reflect the deferred neurotoxic action of 3-HOK on the fly nervous system, leading to calyx synaptic pathology and memory defects in aged *cd^1^* (Savvateeva et al., 2000). On the contrary, *cn^1^* running speed decreases and run frequency rises relative to *CS*, which may be the inhibitory effects of KYNA on glutamate or acetylcholine neurotransmission.

For 5-day-old flies, running speed is about 5 mm/s, being close to previous data obtained by the same method (Fedotov et al., 2014), but much less than the median speed 15 mm/s in Buridan’s paradigm (Colomb et al., 2012) and similar values published in (Bayne et al., 2005). The differences may be explained by much less space of movement in our experiments, as flies regulate their walking speed, slowing when some objects are nearby (Creamer et al., 2018).

*cd^1^* third instar larvae also show the increase in running speed, while *cn^1^* demonstrated the total decrease in SLA parameters (Zakharov et al., 2012). As the larvae lack compound eyes, the observed effects are not due to defects in xanthommatin synthesis. Male courtship indices are equal for *CS*, *cn^1^*, and *cd^1^*, remaining high during the whole life, indicating that their response to the courtship-regulating pheromones and visual cues from a female remains unchanged. Three-hour memory in conditioned courtship suppression paradigm (CCSP) is intact in old *CS* and *cn^1^*, for *cd^1^* it decreases since day 13 (Savvateeva et al., 2000). All these mutants differ in most SLA parameters on days 13–29, so we do not observe any correlation between their SLA and cognitive abilities in CCSP. Though the observed changes in running speed seem not to depend on the visual system, SLA investigation for kynurenine mutants in the darkness would clarify its effect on flies’ orientation abilities.

Previously, the percentage of 8–20-day-old *cd* running females was shown to be less than for *CS*. Running speed was not estimated there. *cn* was less active than *CS* after day 3, but there was no clear difference between *cn* and *cd* relative to *CS* observed by us. Both *cn* and *cd* had low life expectancy (~42 and 43 days vs. 48 days for *CS*), while *v* with KPTM block had the maximum lifespan (~64 days; Kamyshev, 1980).

The rise of 3HOK/KYNA index, which can be achieved by inhibiting TDO or KMO, correlates with neurodegeneration in *htt* mutant (Campesan et al., 2011; Green et al., 2012). For the *scarlet* mutant with a defect in 3-HOK transport to pigment cells, age-dependent progressive loss of dopaminergic neurons belonging to Protocerebral Posterial Lateral 1 (PPL1) cluster was shown, along with increase in ROS level, shortened lifespan, and decreased climbing index. *cn^1^* also showed lower climbing index and shortened lifespan, but no loss of PPL1 neurons, which seem to be specifically susceptible to 3-HOK-induced neurodegeneration (Cunningham et al., 2018). As we investigated SLA on the horizontal plane, the negative geotaxis did not affect the flies’ activity. Study of PPL1 functioning for the old *cd^1^*, normal and with antioxidants preventing ROS formation, is a task for future.

Run frequency and running speed are independently controlled by *Drosophila* nervous system (Colomb et al., 2012). Flies’ walking is accompanied by global increase in activity of dopamine neurons within the brain (Aimon et al., 2019), that is in agreement with role of PPL1 neurons in movement control (Cunningham et al., 2018). Neural network responsible for generation of insect motor pulses is localized in thoracic segments (Bassler, 1983). SLA decreases after disruption of a network interconnecting the parts of the *Drosophila* central complex (CX; Martin et al., 1999). The decrease in running speed correlates with defects of the protocerebral bridge and some other parts of CX (Strauss and Heisenberg, 1993). At the same time, CX performs the complex sensory integration and pre-motor processing, regulating fly posture, turning, navigation, selection of motor actions, and inducing sleep. The mushroom bodies also control locomotion activity, mostly suppressing it (Emanuel et al., 2020).

Lack of PB-3-HOK in *v^1^* heads is obviously due to KPMT blockade, and its minimal level in *cn^1^* seems to be an effect of the specific 3-HOK synthesis impairment. As *cd^1^* accumulates 3-HOK, in the case of protein non-enzymatic modification by 3-HOK, we would expect an increase in *cd^1^* PB-3-HOK level. The reverse effect lets us to suppose a novel type of enzymatic modification of *Drosophila* proteins by 3-HOK, possibly associated with PHS. This explains the simultaneous decrease in 3-HOK enzymatic dimerization and its conjugation to proteins. Most of PB-3-HOK is located in the head capsules. As we have shown, it is also present in brains. An obvious PB-3-HOK decrease within *CS* Ped proves the specificity of staining. Ped contains the axons of Kenyon cells extending into the lobes of the mushroom body. This structure is known to be crucial for *Drosophila* olfactory learning and memory, but its role in 3-HOK distribution remains enigmatic.

The function of PB-3-HOK in *Drosophila* is unclear. 3-HOK and its derivatives can serve as UV-filters protecting retina (Korlimbinis and Truscott, 2006). Possibly, they play a similar role in the fly tissues. In some insects, xanthommatin forms a complex with an eye protein (Ajami and Riddiford, 1971). In *Drosophila*, the eye pigments are contained in a specific structure within pigment cells called ommochromasome, where PHS is localized (Figon and Casas, 2019). Thus, PB-3-HOK may be an intermediate of some protein-pigments complex in *Drosophila* eye.

Another role of 3-HOK conjugation to proteins may be its withdrawal from the active circulation. 3-HOK level is extremely high in *Drosophila*, reaching 396 μg/g fresh weight for *cd^1^* tissues (Howells et al., 1977) or about 1.7 mM. This is much more than 3-HOK concentration used *in vitro* to induce apoptosis in neuronal cell culture (Okuda et al., 1996). Such a high 3-HOK level is necessary for *Drosophila* to synthesize eye pigments, but it’s excess would be toxic in the absence of a system of 3-HOK deactivation/deposition. Some specific protein carriers or membrane organelles, such as ommochromasomes, may perform this function.

In summary, 3-HOK accumulation seems to have a dual effect on *cd^1^* SLA, enhancing it for the middle-aged flies and lowering it for the old flies. *cd^1^* mutation also shows a dual effect on 3-HOK metabolism, disrupting xanthommatin synthesis, and 3-HOK conjugation to some proteins. In both cases, free 3-HOK should accumulate in *cd^1^* brains leading to neurotoxicity development. Further studies, including PHS gene sequencing and checking the putative PHS involvement in PB-3-HOK formation, are necessary to elucidate the molecular mechanisms of 3-HOK metabolism in *cd^1^*.

## Data Availability Statement

All datasets generated for this study are included in the article/Supplementary Material.

## Author Contributions

AZ designed the study. AZ and PI performed the analysis of spontaneous locomotor activity. AZ and OV performed the analysis of PB-3-HOK level. AZ performed the analysis of PB-3-HOK distribution in *Drosophila* brains. AZ and ES-P wrote sections of the manuscript. All authors contributed to manuscript revision, read, and approved the submitted version.

### Conflict of Interest

The authors declare that the research was conducted in the absence of any commercial or financial relationships that could be construed as a potential conflict of interest.

## Footnotes

^1^https://www.socscistatistics.com
