# Supplementary material for: 3-Hydroxykynurenine in Regulation of Drosophila Behavior: The Novel Mechanisms for Cardinal Phenotype Manifestations
Source: Front Physiol. 2020 Aug 7;11:971. doi: 10.3389/fphys.2020.00971 (PMC7426499; doi:10.3389/fphys.2020.00971)
Supplement: Supplementary file 1 [file Data_Sheet_1.PDF]

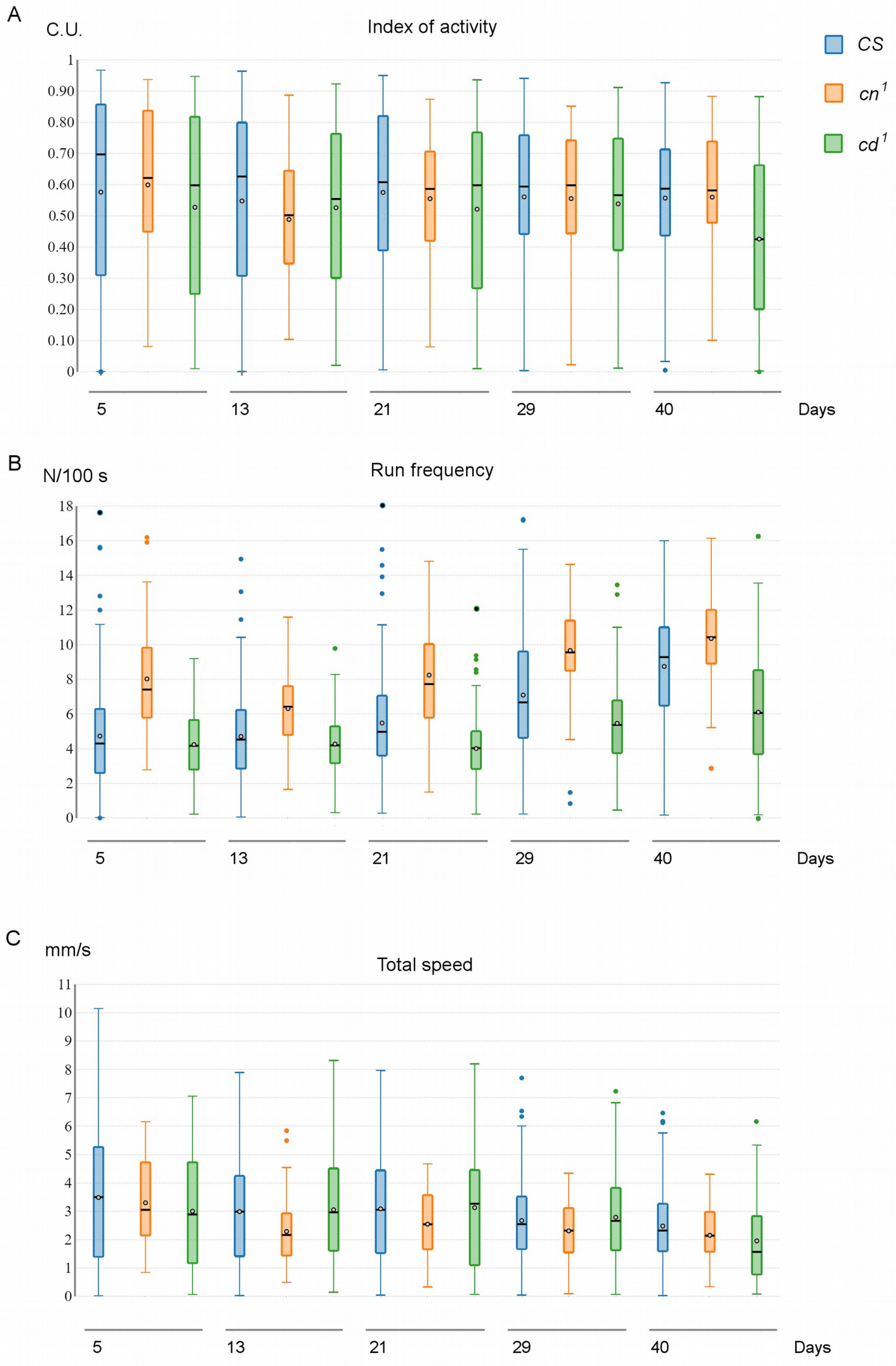
A
C.U.
Index of activity
CS
cn 1
cd 1
1
0.90
0.80
0.70
0.60
0.50
0.40
0.30
0.20
0.10
0
5
13
21
29
40
Days
B
N/100 s
Run frequency
18
16
14
12
10
8
6
4
2
0
5
13
21
29
40
Days
C
mm/s
Total speed
11
10
9
8
7
6
5
4
3
2
1
0
5
13
21
29
40
Days

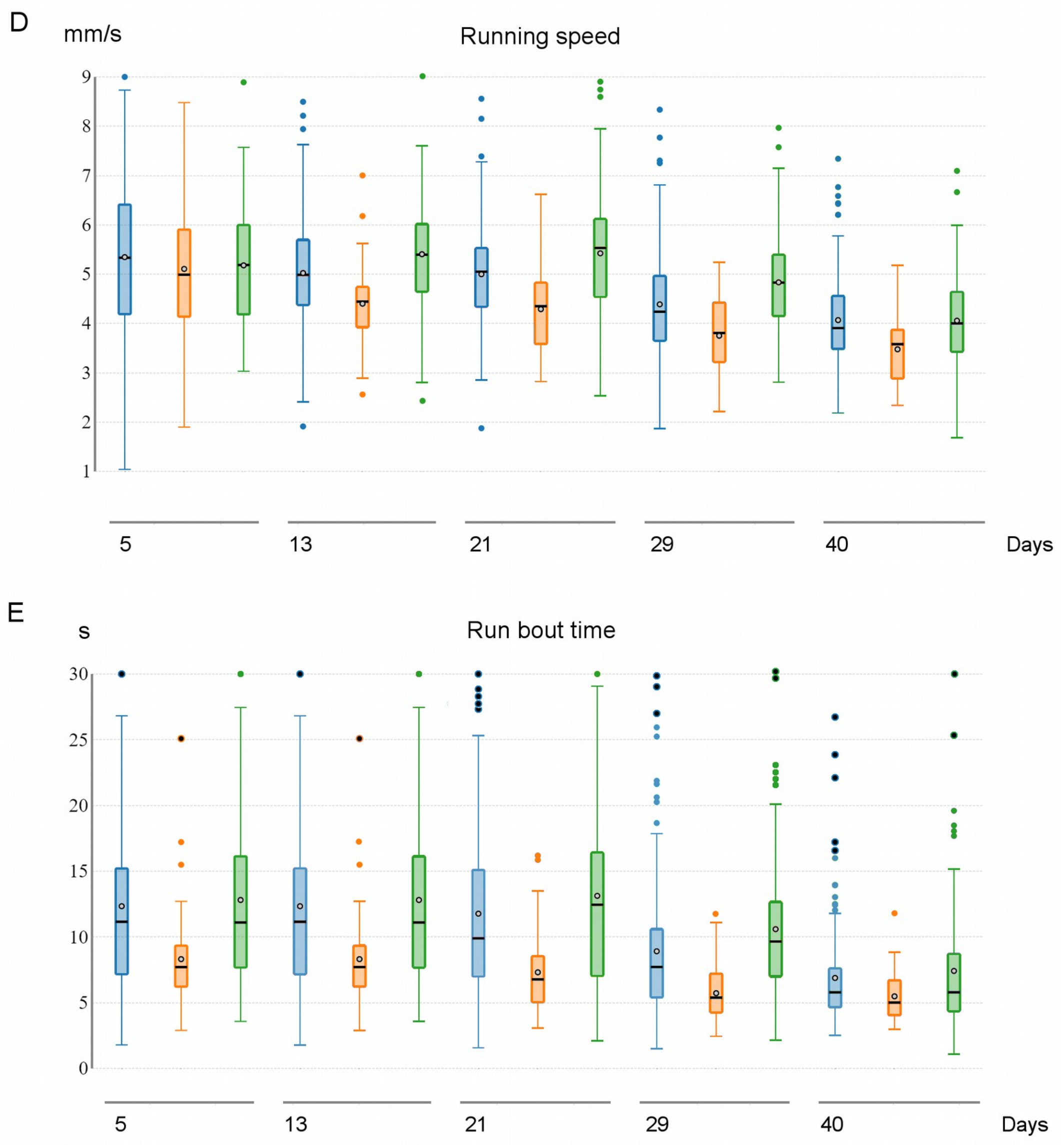

S1 Figure. Box-and-whisker charts for Drosophila SLA parameters.
The charts are made with the help of https://goodcalculators.com/box-plot-maker/.
The mean values are shown as grey circles.

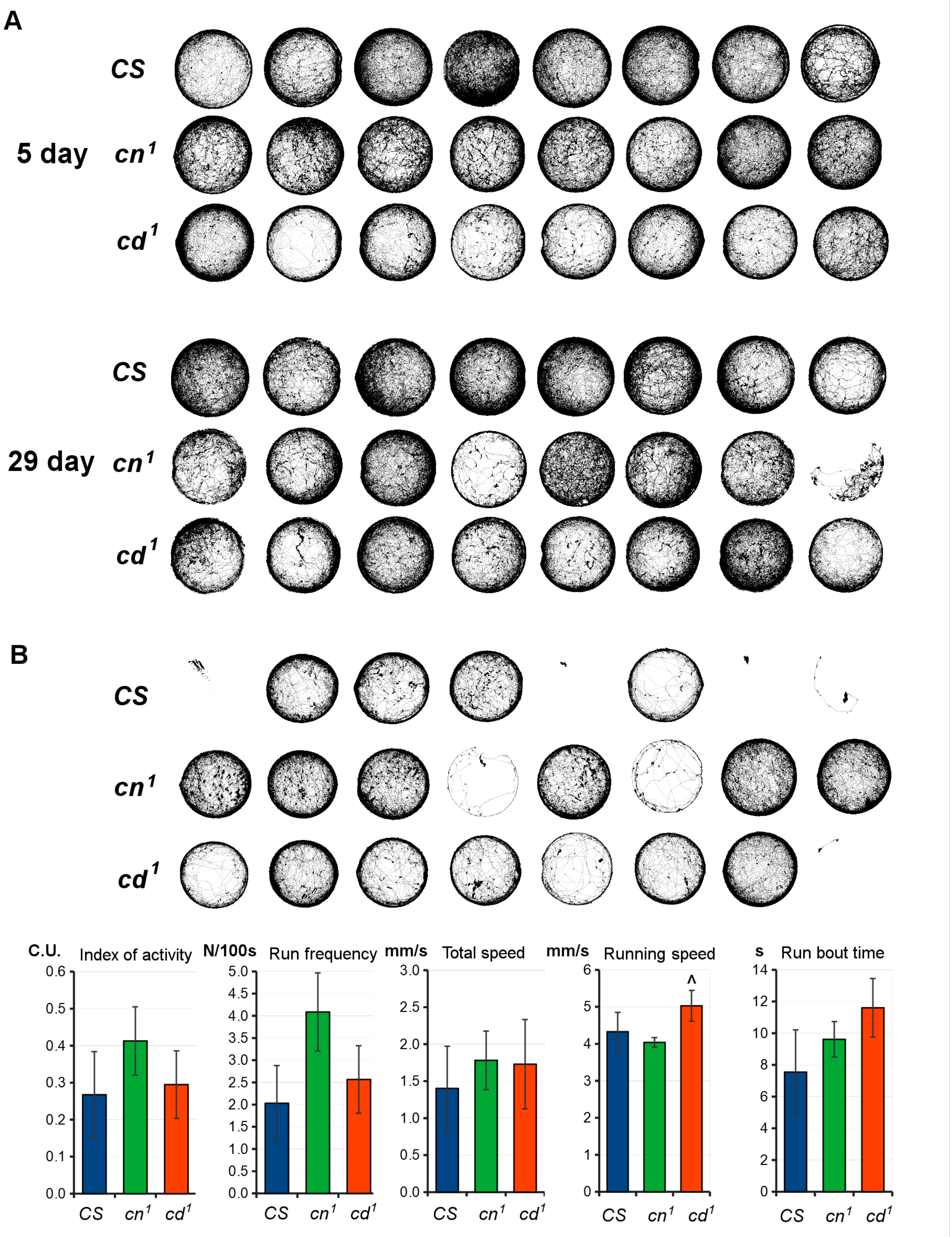

S2 Figure. A. Fly tracks. B. Tracks and SLA parameters for 1-day-old flies studied under red light. ^ *cd*[1] difference from *cn*[1] (two-sided *t*-test; n =8, $p < 0.05$).

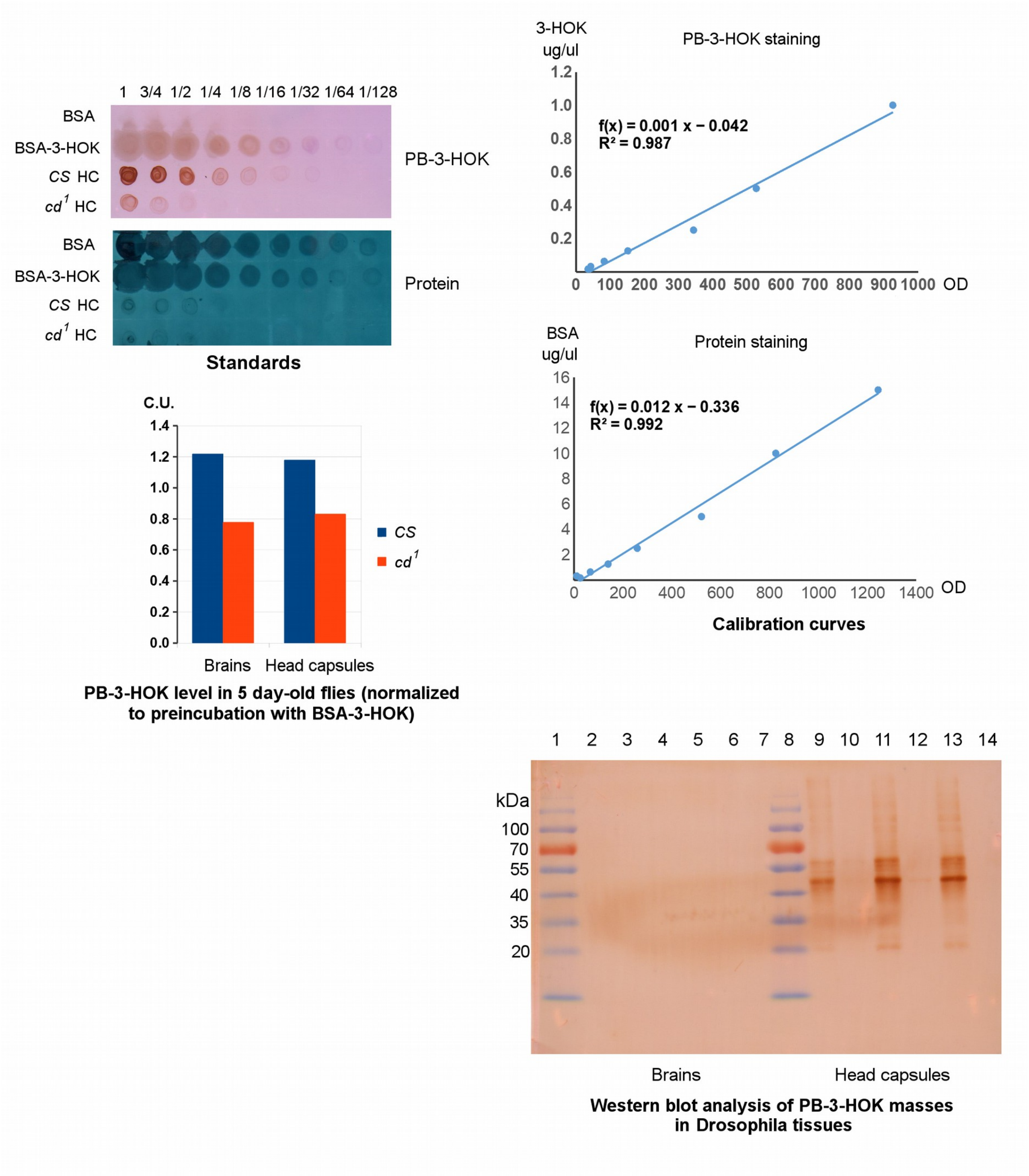

S3 Figure. Dot blot and western-blot analysis of PB-3-HOK in Drosophila brains and head capsules Dot blot analysis: Standards: 1 – 1/128 is a serial dilution, 1X concentrations: 2 ug/ul for 3-HOK used in conjugation with BSA, 20 ug/ul for BSA. PB-3-HOK is 3-HOK conjugated to BSA (BSA-3-HOK) or Drosophila proteins. Protein is total protein stained with amido black. For preincubation, 3.5 ul of antibodies and 10 ul of BSA-3-HOK conjugate were mixed; the difference in PB-3HOK level with and without preincubation is expressed in C.U. OD – integrated optical density, HC – head capsules. Western blot analysis: 1, 8: PageRuler Prestained Protein Ladder (ThermoFisher Scientific, #26616). Molecular masses are shown in kDa. 2 – 7: brains, 9 – 14: head capsules. 2, 9: *CS* 13 day; 3, 10: cd^1 13 day; 4, 11: *CS* 21 day; 5, 12: cd^1 21 day; 6, 13: *CS* 29 day; 7, 14: cd^1 29 day.
